# Metastatic Melanoma With Sebocyte-Like Melanocytes and Widespread Visceral Involvement

**DOI:** 10.5826/dpc.1002a45

**Published:** 2020-04-20

**Authors:** Li-Wei Chang, Viktoryia Kazlouskaya, Jenna R. Bordelon, Marion A. Hughes, Yana G. Najjar, Yuri Bunimovich, Arivarasan Karunamurthy, Jonhan Ho

**Affiliations:** 1 Department of Dermatology, University of Pittsburgh Medical Center, Pittsburgh, PA, USA; 2 Department of Radiology, University of Pittsburgh Medical Center, Pittsburgh, PA, USA; 3 Department of Medicine, University of Pittsburgh Medical Center, Pittsburgh, PA, USA

**Keywords:** sebocyte-like melanocyte, metastatic melanoma, clear cell melanocyte, melanocyte, malignant melanoma

## Introduction

Melanocytes may assume a variety of shapes: epithelioid, round, spindled, pagetoid, dendritic, and with clear cytoplasm, among others. A type of melanocyte with multivacuolated cytoplasm surrounding a dark-stained scalloped nucleus was initially described by Eftychiadis et al as “sebocyte-like” [1]. These melanocytes are more commonly seen in benign congenital nevi, but also have been rarely reported in malignant melanoma, with only a few case reports in the literature [2]. The clear cell change is believed to result from intracellular enlargement of melanosomes with separation of their fibers. Such neoplasms may mimic sebaceous neoplasms and clear cell carcinoma. We report a case of a metastatic melanoma with undifferentiated cells exhibiting sebaceous-like features.

## Case Presentation

**Figure 1:**
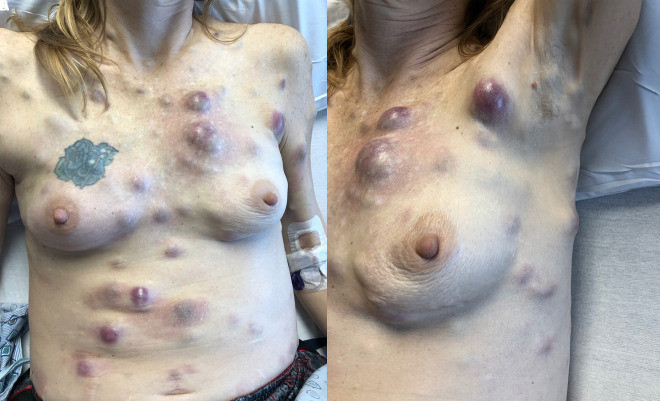
Multiple tender, firm, subcutaneous nodules on the trunk (A) with involvement of left axilla (B).

**Figure 2:**
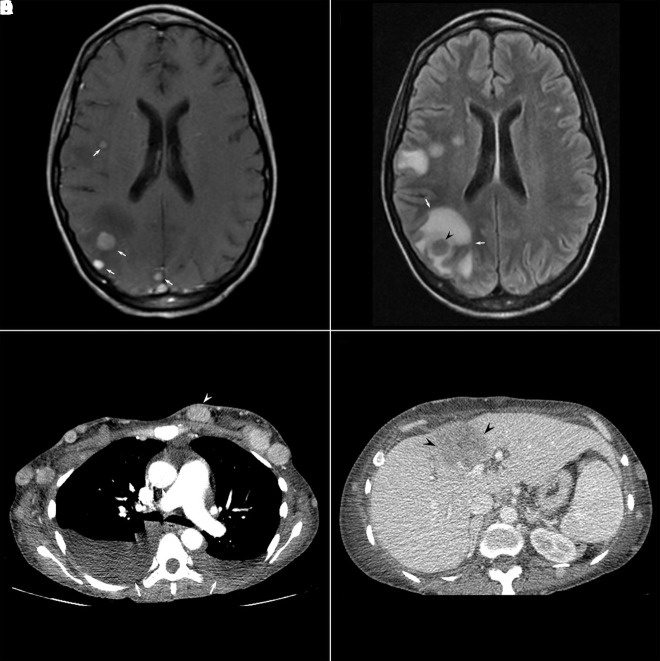
Radiology findings. (A) Axial T1 weighted image with contrast demonstrates multiple enhancing intracranial lesions (white arrows) within the right frontal and parietal lobe consistent with metastases. (B) Axial fluid-attenuated inversion recovery (FLAIR) images demonstrate varying degrees of vasogenic edema (white arrows) surrounding the brain metastases, most notably the right parietal lesion (black arrowhead). (C) Axial CT image of the chest demonstrates multiple subcutaneous metastatic nodules (white arrowhead on a representative nodule). Bilateral pleural effusions (white arrows) are also noted. (D) Axial CT image of the abdomen with contrast demonstrates the large hypoenhancing left hepatic lobe lesion, compatible with a hepatic metastasis (black arrowheads).

A 44-year-old woman with no significant medical history and no family history of melanoma presented with numerous firm subcutaneous nodules on her scalp, trunk, and extremities. The patient first noticed a nodule on her left upper arm 6 months prior, and then similar nodules arose diffusely. The patient endorsed fatigue, dyspnea, dysphagia, epigastric pain, right-sided weakness, and right leg pain. Physical examination revealed numerous 1- to 6-cm, firm, flesh-colored to red subcutaneous nodules diffusely on the scalp, neck, trunk, and extremities (Figure 1). A total body skin examination did not identify lesions of concern for primary malignancy. Brain MRI and a whole-body CT scan revealed widespread soft tissue metastases, with innumerable lesions in the brain, chest wall, liver, spleen, and bone (Figure 2).

**Figure 3:**
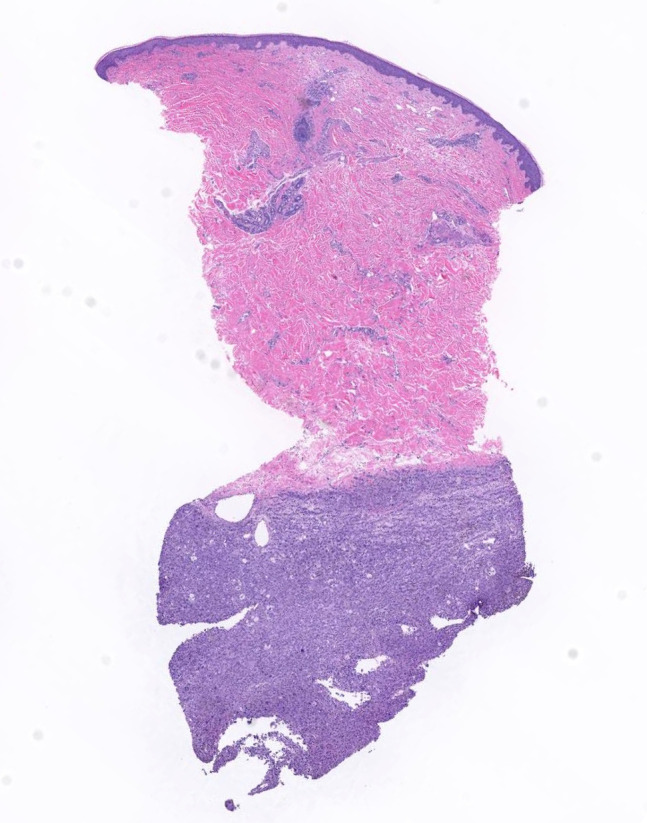
Dermal proliferation with sheets of undifferentiated cells. H&E staining. Scanned slide, cropped image.

**Figure 4:**
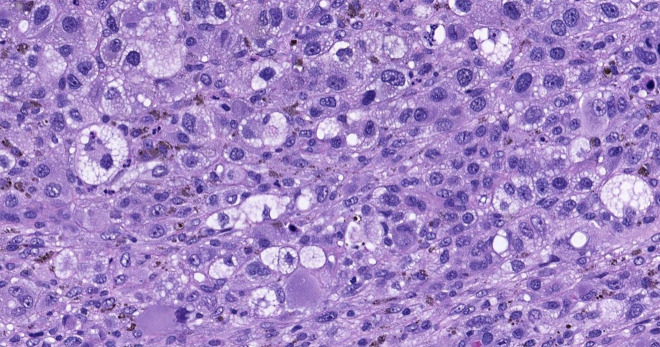
Higher power shows undifferentiated cells with sebo-cyte-like morphology and melanin pigment. H&E staining. Scanned slide, cropped image.

**Figure 5:**
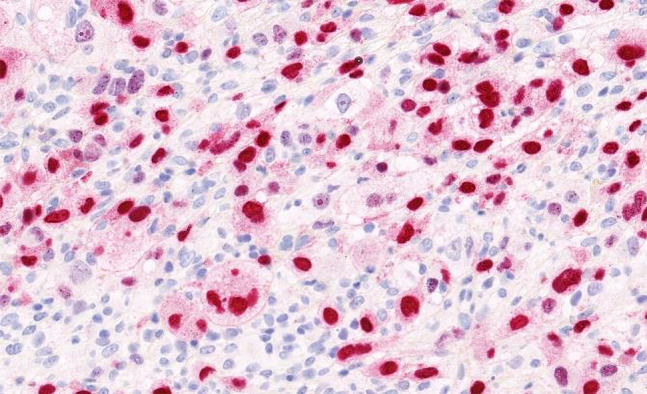
Sebocyte-like cells in the dermal tumor express SOX10. SOX10 immunostaining, cropped image.

Skin biopsies taken from 2 nodules both revealed a deep dermal tumor composed of sheets of densely arranged atypical cells (Figure 3). Morphologically, many cells had scalloped nuclei and “bubbly” cytoplasm resembling sebaceous cells (Figure 4). The cells expressed SOX10 (Figure 5) and were negative for pancytokeratin. *EWSR1* fusion gene study, to rule out clear cell sarcoma, was negative. A diagnosis of metastatic melanoma was made; *BRAF^V600E^* mutation was identified. In addition, the patient had a pericardial effusion requiring a pericardial window and melanoma cells were found in the pericardial fluid. The patient was then started on encorafenib and binimetinib, and within 72 hours her cutaneous lesions were visibly regressing. A subsequent whole-body imaging after 2 months of treatment demonstrated marked reduction in the size and number of subcutaneous nodules in the thoracic and abdominal wall, as well as all visceral lesions. The majority of brain parenchymal lesions also decreased in size. At 4-month follow-up, the patient continued to do well clinically, but brain MRI demonstrated progression of metastatic disease.

## Conclusions

The implication of sebocyte-like features on the prognosis of melanoma is not well established. Few other cases have been reported, and none were as rapidly progressive as in this patient [2]. *BRAF^V600E^* mutation occurs in 50% of melanomas and has historically been associated with more aggressive disease. Treatment with combined BRAF and MEK inhibitors carries a high response rate and is therefore often first-line in those with high disease burden. We present a case of melanoma with sebocyte-like features that carries *BRAF^V600E^* mutation and significantly responded to combination targeted therapy.
